# Clinical presentation of scabies from 1996 to 2022: a retrospective cohort study from Finland

**DOI:** 10.1080/02813432.2025.2511071

**Published:** 2025-06-01

**Authors:** Anna Mikola, Ella Jokela, Jari Jokelainen, Eetu Kiviniemi, Suvi-Päivikki Sinikumpu, Laura Huilaja

**Affiliations:** ^a^ Faculty of Medicine, University of Oulu, Oulu, Finland; ^b^ Northern Finland Birth Cohorts, Arctic Biobank, and Infrastructure for Population Studies, University of Oulu, Oulu, Finland; ^c^ Department of Dermatology, Oulu University Hospital, and Research Unit of Clinical Medicine, University of Oulu, Oulu, Finland

**Keywords:** Scabies, epidemiology, infestation, pruritus, itch

## Abstract

**Background:**

Increasing numbers of scabies cases have been reported in Europe and around the world in recent years. Scabies is more common in children and adolescents than in adults.

**Objectives:**

To characterise patient profiles and treatment strategies of scabies over time at the dermatology clinic of Oulu University Hospital (OUH), Finland.

**Methods:**

The OUH database was searched using diagnostic codes, and all patients diagnosed with scabies in the OUH dermatology unit between 1996 and 2022 were included in the study. The retrieved patient records were reviewed for demographic and clinical data.

**Results:**

The study included the records of 662 patients. ‘Suspected scabies’ was given as reason for referral to a specialist care in only 21.5% of cases. Time between a symptom onset and the referral to the dermatology unit was significantly longer in adults and adolescents than in small children (*p* < 0.001), and was longer than six months in one-fifth of adults. Symptomatic sites varied between age groups: compared with small children, adults more commonly (*p* < 0.001) presented with symptoms in the genital-gluteal area, whereas symptoms were located on the head and ‘foot and ankles’ more often in young children than other age groups (*p* < 0001 for all comparisons).

**Conclusions:**

Diagnosis was markedly delayed in many patients, especially adults. Unspecified, pruritic skin symptoms should lead to a suspicion of scabies, and when a diagnosis is unclear, the patient should be referred to a dermatologist as quickly as possible. This would both help patients and prevent further spread of the infestation.

## Introduction

Scabies is a parasitic skin infestation caused by *Sarcoptes scabiei var. hominis.* It spreads mostly by direct skin-to-skin contact and rarely indirectly by contact with contaminated materials [1]. The risk for spreading increases in relation to the number of parasites present and the duration of contact [2]. After approximately 4–6 weeks, infestation is followed by intense itching, which results from delayed hypersensitivity reaction to various mite-related antigens [1,3].

Scabies is a globally significant condition and a growing public health issue. The burden is particularly high in Asia, Oceania and in tropical Latin America [4,5]. Reports have described increasing numbers of scabies cases in several European countries, especially over the past two decades [6–9]. Despite recent updates in scabies epidemiology globally, there is still a call for more data especially from Europe and from Northern America [10]. Although these are high-income areas in general, recent outbreaks of scabies [6–9] highlight the need of up-to-date information as epidemiology may change. This has been well demonstrated in studies conducted in Italy and Turkey, which reported markedly and rapidly increased incidence of scabies shortly after COVID-19 pandemic [11,12] We recently reported a significant increase in the incidence of scabies for two decades period and the incidence increased particularly among adolescents [13].

In Finland, public primary and secondary care is government funded for every individual living in Finland. Everyone can contact primary health care according to their needs for example when having skin symptoms or itch. Over-the-counter medications are available for self-medication of scabies as well, and some may seek help from a private practicing physician or dermatologist by their own cost. Based on primary care physician’s or private practitioner’s evaluation, an individual can be referred to the hospital dermatologist if needed. In the present study, we aimed to analyse the demographic and clinical characteristics, diagnostic delays, and the treatments that were prescribed to patients diagnosed with confirmed or clinical scabies in the dermatology unit of Oulu University Hospital (OUH), Finland, over a period of 26 years.

## Materials and methods

OUH serves as secondary referral centre for around 410,000 individuals. OUH database was searched to obtain the records of all patients diagnosed with scabies at the OUH dermatology unit from Jan 1^st^ 1996 to December 31^st^ 2022. Each case was identified by the presence in the patient’s electronic record of the International Classification of Diseases, Tenth Revision (ICD-10) code B86. Study period was chosen to begin from 1996 as electronic patient records (EHR) including all in- and out-patient cases were available from 1996 onwards. Records were reviewed by two of the investigators (EJ and AM) to extract relevant demographic and clinical data. Records not containing evidence that the diagnosis had been verified either by a microscopic identification of the mite, a dermoscopy, or by an observation of clinical presentation typical of scabies were excluded from the study [2]. Each patient was categorised by age group as follows: ‘small children’ (<5 years old), ‘children’ (≥5 to <15 years old), ‘adolescents’ (≥15 to <20 years old) and ‘adults’ (>20 years old).

All statistical analyses were performed using the R software package (version 4.3.0, R-Foundation for Statistical Computing, Vienna, Austria). Results are presented as proportions, means and standard deviation (SD). *P* values ≤0.05 were considered statistically significant.

The study was approved by the Medical Director of OUH. As this was a retrospective registry study, no approval by an ethical review board was required under Finnish legislation.

## Results

The database search for the ICD-10 code for scabies returned the records of 709 patients. After 47 exclusions as described above, the final study population included a total of 662 verified scabies cases, 47.7% were female and the mean (SD) age was 30.2 (20.3) years. Slightly more than half (52.6%) of the cases were adults, and the remainder children or adolescents (≤20 years old) at the time of the diagnosis.

As shown in Table 1, the most common reasons for referral to the dermatology unit in the overall study population were ‘unspecified dermatitis/skin symptoms’, pruritus and a suspicion/diagnosis of scabies itself. Small children were significantly more often referred with scabies suspicion than adults (*p* < 0.001) or adolescents (*p* < 0.001). Pruritus was a less common reason for referral of small children than adults (*p* < 0.001).

**Table 1.** Characteristics of study population.

	All	Small children	Children	Adolescents	Adults	P value
**N (%)**	662 (100.0)	80 (12.1)	95 (14.4)	139 (21.0)	348 (52.6)	
**Female**	316 (47.7)	36 (45.0)	60 (63.2)	67 (48.2)	153 (44.0)	0.010
**Mean age, years**	30.2	1	10	18	47	
**Reason for referral**						
*Unspecified dermatitis or unspecified skin symptoms*	264 (39.9)	30 (37.5)	35 (36.8)	57 (41.0)	142 (40.8)	0.861
*Pruritus*	156 (23.6)	4 (5.0)	16 (16.8)	36 (25.9)	100 (28.7)	<0.001
*Scabies*	142 (21.5)	36 (45.0)	28 (29.5)	25 (18.0)	53 (15.2)	<0.001
*Specified dermatological diagnosis* ^a^	64 (9.7)	6 (7.5)	10 (10.5)	12 (8.6)	36 (10.3)	0.834
*Suspicion of venereal infection*	11 (1.7)	0 (0)	0 (0)	3 (2.2)	8 (2.3)	0.340
*Other* ^b^	7 (1.1)	1 (1.3)	2 (2.1)	2 (1.4)	2 (0.6)	
*Scabies in family*	2 (0.3)	0 (0)	0 (0)	0 (0)	2 (0.6)	
*Unknown*	16 (2.4)	3 (3.8)	4 (4.2)	4 (2.9)	5 (1.4)	
**Known previous scabies infestation in history**	27 (4.1)	5 (6.3)	3 (3.2)	8 (5.8)	11 (3.2)	0.354
**Duration of symptoms**						<0.001
*≤1 month*	69 (10.4)	17 (21.3)	11 (11.6)	14 (10.1)	27 (7.8)	
*1–2 months*	190 (28.7)	34 (42.5)	27 (28.4)	42 (30.2)	87 (25.0)	
*>2–6 months*	256 (38.7)	15 (18.8)	37 (38.9)	55 (39.6)	149 (42.8)	
*≥6 months*	119 (18.0)	8 (10.0)	16 (16.8)	24 (17.3)	71 (20.4)	
*Unknown*	1 (0.2)	0 (0)	0 (0)	0 (0)	1 (0.3)	
**Symptom sites** ^c^						
*Extremities, any location*	640 (96.7)	78 (97.5)	94 (98.9)	137 (98.6)	331 (95.1)	0.207
*Wrists, hands*	584 (88.2)	61 (76.2)	91 (95.8)	124 (89.2)	308 (88.5)	0.001
*Armpits*	36 (5.4)	4 (5.0)	4 (4.2)	5 (3.6)	23 (6.6)	0.606
*Antecubital area*	119 (18.0)	13 (16.3)	18 (18.9)	27 (19.4)	61 (17.5)	0.926
*Foot, ankles*	253 (38.2)	57 (71.3)	33 (34.7)	51 (36.7)	112 (32.2)	<0.001
*Lower limb (other)*	129 (19.5)	7 (8.8)	20 (21.1)	28 (20.1)	74 (21.3)	0.080
*Trunk*	452 (68.3)	56 (70.0)	56 (58.9)	87 (62.6)	253 (72.7)	0.027
*Buttocks, genitals*	174 (26.3)	8 (10.0)	11 (11.6)	35 (25.2)	120 (34.5)	<0.001
*Head, neck*	53 (8.0)	24 (30.0)	6 (6.3)	5 (3.6)	18 (5.2)	<0.001
*Breasts*	13 (2.0)	0 (0)	1 (1.1)	4 (2.9)	8 (2.3)	0.515
*Not specified*	7 (1.1)	2 (2.5)	0 (0)	1 (0.7)	4 (1.1)	
**Comorbid dermatologic condition**	124 (18.7)	14 (17.5)	18 (18.9)	30 (21.6)	62 (17.8)	
*Atopic dermatitis*	62 (9.4)	13 (16.3)	11 (11.6)	12 (8.6)	26 (7.5)	
*Other eczema*	23 (3.5)	1 (1.3)	1 (1.1)	2 (1.4)	19 (5.5)	
*Psoriasis*	7 (1.1)	0 (0)	0 (0)	0 (0)	7 (2.0)	
*Urticaria*	4 (0.6)	0 (0)	3 (3.2)	0 (0)	1 (0.3)	
*Other* ^d^	28 (4.2)	0 (0)	3 (3.2)	16 (11.5)	9 (2.6)	
**Origin of the infestation**						0.011
*Not identified or information not recorded in records*	608 (91.8)	67 (83.8)	86 (90.5)	134 (96.4)	321 (92.2)	
*Identified*	54 (8.2)	13 (6.2)	9 (5.5)	5 (3.6)	27 (7.8)	

All data are N (%).

^a^ Other than scabies (i.e. worsening of atopic dermatitis, lichen planus, dermatitis herpetiformis).

^b^ I.e. as a secondary diagnosis at control visit due to another dermatological condition.

^c^ All symptomatic areas/case were included.

^d^ Includes two cases with acne and two with folliculitis. Other diagnoses were found only in one case.

Small children were referred to the dermatology clinic significantly earlier after symptom onset than children (*p* = 0.018), adolescents (*p* = 0.003) or adults (*p* < 0.001) 63.8% of them were referred within two months of symptom onset. More than half of the adults (63.2%) and adolescents (56.9%) had had symptoms longer than three months by the time of their referral. One-fifth (20.4%) of the adults were diagnosed more than six months after the appearance of symptoms. Almost one-fifth (18.7%) of all cases had another skin condition as a comorbidity, atopic eczema being the most common in all age groups. In 91.8% of the cases the source of infestation was unknown/uncertain (Table 1). The most frequent known source was within the patient’s family (87.0%).

The most common area for skin symptoms was ‘wrists, hands’ in all age groups. Adults had symptoms more commonly in ‘buttocks or genitals’ compared to small children (34.5% vs 10.0%, *p* < 0.001) and children (11.6%, *p* < 0.001). Head (30.0%) and ‘foot and ankles’ (71.2%) were found to be significantly more often symptomatic in small children when compared to other age groups (*p* < 0.001; Table 1)

The source of 54.4% of all referrals was primary healthcare, 14.8% were from other hospital departments, and 6.9% from private health care (including GPs and all specialties). For a fifth (19.8%), the referring unit could not be retrospectively defined as only referring doctor’s name was recorded in EHR. Small children were more commonly sent for consultation within the hospital (36.2.%) than children (20.0%), adolescents (10.1%) or adults (10.3%) (*p* < 0.001, for small children vs. other age groups).

**Figure 1.**
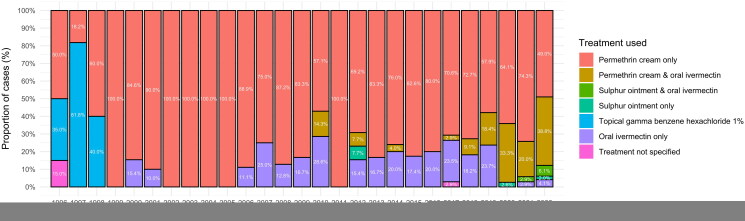
Treatments by year.

The most common treatment in all age groups was topical permethrin (Table 2). From 2018 onwards, there has been a growing trend towards a treatment with a combination of topical permethrin and oral ivermectin (Figure 1).

**Table 2.** Treatments by age group.

	All	Small children	Children	Adolescents	Adults
**N**	662	80	95	139	348
**Treatment used, N (%)**					
Permethrin cream only	502 (75.8)	72 (90.0)	72 (75.8)	105 (75.5)	253 (72.7)
Permethrin cream & oral ivermectin^a^	54 (8.2)	2 (2.5)	6 (6.3)	12 (8.6)	34 (9.8)
Sulphur ointment & oral ivermectin^a^	4 (0.6)	0 (0)	2 (2.1)	1 (0.7)	1 (0.3)
Sulphur ointment	3 (0.4)	3 (3.8)	0 (0)	0 (0)	0 (0)
Oral ivermectin^a^ only	74 (11.2)	1 (1.3)	9 (9.5)	16 (11.5)	48 (13.8)
Topical gamma benzene hexachloride 1%^b^	20 (3.0)	1 (1.3)	6 (6.3)	5 (3.6)	8 (2.3)
Treatment not specified	4 (0.6)	1(1.3)	0 (0)	0 (0)	3 (0.9)

^a^ Available for oral human use in Finland from January 2006.

^b^Available in Finland until June 1998.

## Discussion

During our study period, the most cases diagnosed with scabies were adults (>20 years old, with mean age of almost fifty years), while the Dutch study found the highest incidence among 20–24-year-olds [7]. As our study period is twice longer than in the Dutch study, the longer time period in conjunction with recent changes in age-specified [13] incidence most likely explain this difference. Interestingly, other European studies found the highest incidence among comparatively young age groups: in Spain, the predominant group was <15 years old [6] and in the United Kingdom, it was 10–19 years old [14]. This difference may at least partially be explained by differences in the data sources as both the UK and Spanish studies used data from cases diagnosed mainly in the primary care setting. However, in global context, scabies more commonly affects children than adults [5].

We found that adolescents and adults tended to have a relatively long delay from symptom onset to diagnosis: in more than half, the delay was longer than three months, and in a fifth of adults, more than six months. These delays are markedly longer than the mean diagnostic delay of 62 days reported by a study conducted in French dermatology departments (*N* = 323) [15]. However, most (*N* = 199) of the cases in the French study were ≤15 years old, which may have affected the findings. With regard to our sub-population of small children, 63.8% had their diagnosis within two months of symptom onset, a finding which is comparable to that of the French study [15]. Our finding of a significant difference in diagnostic delay between age groups most likely reflects the difficulty of confirming a scabies diagnosis, since there are numerous differential diagnoses to consider [3]. In our study, patients were most commonly (63.5%), referred to OUH with either unspecified skin symptoms or pruritus. Our data suggest that diagnosing scabies may be easier in small children, as this age group was referred with a diagnosis/suspicion of scabies significantly more often than the other age groups.

Distribution of scabies lesions are known to vary by age [3]. Similarly to the previous literature [3], we found that ‘wrists and hands’ were the most common symptom site in all age groups, and ‘foot and ankles’ and ‘head’ were significantly more often symptomatic in small children than in other age groups. The French study mentioned above found that an armpit involvement was common in children <2 years old [15], but we found no differences between age groups for involvement of this site. In our study, genital symptoms were significantly more common in adults than in small children or children. Contrary to our findings, the French study reported that approximately half of all cases, regardless of age group, had symptoms in genital area or buttocks [15].

Topical permethrin was the most commonly used treatment in all our study’s age groups. The use of oral ivermectin as a monotherapy was constant between 2006 and 2019 (aside from 2011); the use of combination therapy with oral ivermectin and topical permethrin has increased since 2020. A similar growing trend in the numbers of ivermectin prescriptions has been reported in Norway from 2014–2018 [8] and in the Netherlands from 2015 onwards [7]. A concern has been raised that permethrin resistance in scabies mites may have led to increased use of combination regimens [1]. A German study [16] found that an increase in combination therapy was driven by treatment adherence issues, and this may also have been the case in our population. Further studies in Northern Europe are needed to analyse if true resistance to scabicides occurs or if the increase in incidence of scabies is more related to “pseudo-resistance” phenomenon (i.e. underdosing/early-discontinuation, suboptimal adherence).

The main strength of this study is that the analysis included all patients diagnosed with scabies at OUH over a period of more than two decades, and that all diagnoses were made or confirmed by a dermatologist, ensuring the reliability of the diagnoses. Every person living in Finland is eligible for public healthcare, so our data are not limited by the socio-economic status. However, although we included only cases whose records contained entries for ‘confirmed’ or ‘clinical’ scabies [3] the retrospective nature of our study prevented us from verifying the diagnoses and not all cases of scabies in the area during study time were included, since not those diagnosed by GPs were referred to OUH. Based on the publicly available data about scabies diagnoses made in the primary health care, we estimate that yearly number of scabies diagnosed in dermatology department varies from 10% to 36% of all scabies diagnosed in OUH area. The retrospective design also made it inevitable that some data were missing, and we were unable to analyse the treatment outcomes because the records lacked information on follow-up visits.

In summary, despite recent increases in the awareness of scabies, diagnostic delays remain long, especially among adults and adolescents. To ensure early detection, there appears to be an unmet need for greater collaboration, awareness and training resources, among the general population, frontline healthcare professionals and dermatologists. As scabies epidemiology seems to be changing, it is important to continuously monitor numbers of scabies cases to timely tackle its outbreaks.

## Data Availability

Data are available upon request from the Oulu University Hospital. According to Finnish legislation, restrictions apply.
